# After Round Comprehensive Plan Summary tool: an efficiency approach for the ICU

**DOI:** 10.1186/cc14597

**Published:** 2015-03-16

**Authors:** R Marktanner, M Mostafa, A Shafei, H Hon, R Ashraf, P Sreedhara, N Syed, D Gharaibeh, A Taha

**Affiliations:** 1 Sheikh Khalifa Medical City, Abu Dhabi, United Arab Emirates

## Introduction

ICU workflow for physicians, nurses and other healthcare providers is often complicated by distractions, interruptions, difficulties to concentrate, multitasking and the need to frequently change priorities in patient care-related procedures. It is therefore a continuous challenge to communicate and implement plans in an early, efficient and reliable way within the team and to constantly keep track about tasks that are completed, postponed or that are about to be forgotten. To facilitate every caregiver's workflow and to early and efficiently communicate, we implemented the After Round Comprehensive Plan Summary (ARCoPS) tool.

## Methods

Most of our patient care-related therapeutic and diagnostic decisions are made during the morning round consisting of intensivists, nursing team, surgeons, respiratory therapists, dieticians, clinical pharmacists and physiotherapists. Immediately thereafter, all plans for the next 24-hour period are again discussed, summarized, confirmed, prioritized and organized by the multidisciplinary team under aspects of optimal patient care and workflow efficiency by entering the plan data into a flat screen visualized template connected to our intranet immediately accessible at every physician and nursing caregiver workstation.

## Results

Twelve months after implementation of the ARCoPS tool we conducted caregiver interviews and identified the following effects: increase in treatment confidence through standardized multidisciplinary decision-making; reduced loss of information through immediate plan summarization by the whole team; reduction of ambiguity and misunderstanding about care plans through early written documentation; higher level of security not to forget procedures or tasks; positive acceptance of the tool to flexibly change priorities of care procedures; positive acceptance of the tool to mark accomplished tasks providing visual feedback about the care plan status; individual caregiver workflow economization through permanent treatment plan availability; and higher job satisfaction throughout the caregiver team.

## Conclusion

ARCoPS tool utilization has become a daily routine in our ICU. It functions as an effective communication and workflow tool and has helped us to reduce patient care-related misunderstandings and delays. It also enhanced the economics of our work sequence, which also highly contributes to a better level of patient safety. Furthermore, it has markedly contributed to an improved level of quality of work for caregivers.

